# Low Dosage of ABA Enhances Arbuscule Formation and Recovers the Inhibitory Effect of Low pH on This Process

**DOI:** 10.1002/pei3.70124

**Published:** 2026-04-01

**Authors:** Xiaodi Liu, Zengwei Feng, Wei Zhang, Ning Wang, Qing Yao, Honghui Zhu

**Affiliations:** ^1^ Key Laboratory of Agricultural Microbiomics and Precision Application (MARA), Guangdong Provincial Key Laboratory of Microbial Culture Collection and Application, Key Laboratory of Agricultural Microbiome (MARA), State Key Laboratory of Applied Microbiology Southern China Institute of Microbiology, Guangdong Academy of Sciences Guangzhou China; ^2^ Guangdong Province Key Laboratory of Microbial Signals and Disease Control College of Horticulture, South China Agricultural University Guangzhou China; ^3^ Guangdong Polytechnic of Water Resources and Electric Engineering Guangzhou China

**Keywords:** abscisic acid, arbuscular mycorrhizal fungi, arbuscule formation and functionality, low pH

## Abstract

Arbuscular mycorrhizal (AM) fungi can form symbiotic associations with plants and play a significant role in enhancing plant tolerance to acidic stress, wherein arbuscules serve as key structures in this process. However, the response patterns of arbuscule development and function under low pH conditions remain poorly understood. Previous studies have shown that abscisic acid (ABA) can regulate arbuscule development, but whether ABA regulates arbuscule development and function under low pH conditions is unknown. In this study, the model plant tomato ( *Solanum lycopersicum* ) was used as the host plant, inoculated with AM fungi to investigate the regulatory effects of low pH and exogenous ABA on arbuscule development and function. The results showed that (1) as time progressed, the mycorrhizal colonization increased, and arbuscules gradually developed from the main trunk to mature and senescent stages; however, low pH values inhibited arbuscule development. (2) High concentrations of ABA inhibited root growth and mycorrhizal colonization, whereas low concentrations promoted mycorrhizal colonization, with 10^−7^ M identified as the optimal concentration for maximizing mycorrhizal colonization. (3) Both low pH and ABA‐deficient mutants significantly inhibited mycorrhizal colonization, alkaline phosphatase activity, and the expression of genes related to arbuscular development. However, exogenous ABA did not significantly affect the expression of genes associated with arbuscular function. Low concentrations of ABA could restore the inhibition of arbuscule development and function caused by low pH and ABA‐deficient mutants. Additionally, low pH significantly inhibited the ABA content in mycorrhizae, while exogenous ABA treatment significantly increased the ABA content in mycorrhizae. Our research results indicate that low dosage of ABA enhances arbuscule formation and function, and recovers the inhibitory effect of low pH on this process. Low pH may regulate arbuscule development and function by modulating ABA in roots, and ABA may regulate mycorrhizal development by affecting lipid synthesis and transport.

## 1 Introduction

Acidic soil constitutes an important global land resource (Baligar et al. 2004). However, its low pH and other unfavorable conditions negatively affect crop root growth and nutrient uptake, ultimately inhibiting crop yield and quality (Duan et al. 2024, 2025). Arbuscular mycorrhizal (AM) fungi are ubiquitous soil fungi that form obligate symbiotic associations with plants (Rosling et al. 2024). After the establishment of the symbiotic relationship between AM fungi and plants, the resistance of plants to pH stress can be greatly improved, thus promoting plant growth, and it has great application potential in acidic soil (Bhupenchandra et al. 2024; Nie et al. 2024; Ahmed et al. 2025).

Plant hormones, acting as signaling molecules, play a crucial role in regulating the symbiosis between plants and AM fungi (Wang et al. 2024). Abscisic acid (ABA) is a signaling molecule enabling plants to respond to abiotic stress and plays an essential role in regulating the colonization process of AM fungi (Herrera‐Medina et al. 2007; Chen et al. 2024; Liu et al. 2024). For instance, in the *sitiens* mutant of tomato, which exhibits impaired ABA biosynthesis, mycorrhizal colonization, arbuscule formation, and functionality are severely suppressed (Martín‐Rodríguez et al. 2011), while exogenous application of ABA can partially restore AM colonization and arbuscule development in this mutant (Aroca et al. 2008). These findings suggest that ABA positively modulates processes associated with plant‐AM symbiosis (Ahmed et al. 2025; Kandalgaonkar and Barvkar 2025). Furthermore, Charpentier et al. (2014) elucidated the impact of ABA concentration on AM colonization: low ABA concentrations (5 μM) enhance mycorrhizal colonization, whereas high ABA concentrations (50 μM) significantly suppress the colonization process (Charpentier et al. 2014). In summary, the optimal level of ABA facilitates processes linked to plant‐AM symbiosis, while excessive ABA inhibits mycorrhizal colonization and development, indicating that the regulation of AM symbiosis by ABA is concentration‐dependent.

Additionally, Fiorilli et al. (2009) identified 6 genes exhibiting specific expression in cells containing arbuscules, which are involved in auxin and ABA hormone metabolism. This suggests the potential involvement of auxin and ABA in arbuscule formation and/or function. ABA is a plant hormone closely associated with plant aging and has also attracted interest for its role in microbial processes. For instance, ABA can be synthesized by certain plant growth‐promoting rhizobacteria to enhance plant stress tolerance, and it may influence the colonization and biofilm formation of microorganisms on plant surfaces (Chieb and Gachomo 2023; Das and Sarkar 2024). Sodium tungstate (Na_2_WO_4_) is widely recognized in the fields of plant physiology and molecular biology as a specific inhibitor of ABA biosynthesis (Lee and Milborrow 1997; Du et al. 2025). In the symbiosis between AM fungi and tomato plants, application of Na_2_WO_4_ significantly reduced both arbuscular colonization and overall infection rates (Charpentier et al. 2014). This suggests that the decrease in arbuscular colonization may be attributed to the reduction in total mycorrhizal colonization. However, in the symbiotic system between AM fungi and the ethylene‐insensitive mutant of tomato (Rodriguez et al. 2010), Na_2_WO_4_ significantly decreased arbuscular colonization rates without affecting overall mycorrhizal colonization. These findings clearly indicate that ABA plays a regulatory role in arbuscular development by AM fungi (Jing et al. 2025; Vergara and Araujo 2024). The study conducted by Herrera‐Medina et al. (2007) demonstrates the pivotal role of ABA in both the development and functionality of AM fungal branches.

Arbuscules are the core structure for nutrient exchange between both AM fungi and plants, which continuously undergo branching within cortical cells, thereby creating a substantial surface area, while plant root cells continually invaginate to form arbuscular periarbuscular membranes (Feng et al. 2020), facilitating extensive exchanges of carbon and phosphorus nutrients (Vergara and Araujo 2024; Duan et al. 2024). During the symbiosis between AM fungi and plants, the extensive transfer of membrane materials is a critical step in the formation of arbuscular structures (Fukuda et al. 2024; Serrano et al. 2024). Notably, *EXO70I*, as a core component of the exocytosis complex, plays an essential role in the development of periarbuscular membrane (PAM) branches (Zhang et al. 2015). Research has demonstrated that arbuscular development in *EXO70I* mutant plants is significantly impaired. Wang et al. (2012) were the first to demonstrate that the plant gene *RAM2* is essential for arbuscule formation. This gene encodes 3‐phosphoglycerol acyltransferase, which plays a critical role in lipid synthesis within the endoplasmic reticulum. Moreover, *RAM2* facilitates the transfer of lipids from plants to AM fungi or pathogenic fungi (Wang et al. 2012; Park et al. 2015; Li et al. 2024). *STR2* encodes an ATP‐binding cassette transporter located in the periarbuscular membrane, and this protein is crucial for arbuscule development (Zhang, Yin, et al. 2024; Zhang, Zhou, et al. 2024; Ivanov and Harrison 2024). Jiang et al. (2017) further confirmed that *STR* and *STR2* are responsible for transporting host‐synthesized lipids to AM fungi. These findings confirm the pivotal role of lipids in arbuscule formation (Xue et al. 2015).

The environment in acidic soil is rather complex, encompassing not only the reduction of soil pH but also the elevation of aluminum ion concentration and the exacerbation of heavy metal ion toxicity (Shaaban 2024; Mosley et al. 2024; Nilsson 2024). To minimize interference from other environmental factors, we employed MSR medium to establish a sterile culture system for investigating the specific effects of low pH conditions on arbuscular mycorrhizal development. Furthermore, the regulatory mechanism by which ABA influences arbuscular development and function under low pH stress remains incompletely understood. In this study, ABA‐deficient mutants and wild type were inoculated with spores of the arbuscular mycorrhizal fungi *Rhizophagus irregularis* DAOM197198 to examine the relationship between mycorrhizal development and ABA metabolic levels at various time points, thereby elucidating the response pattern of ABA during the symbiotic interaction between plants and AM fungi under low pH conditions.

## 2 Materials and Methods

Tomato was utilized as the plant material, including ABA‐deficient mutants and their corresponding wild types, which were procured from the Tomato Genetics Resource Center (TGRC) seed repository at the Department of Plant Sciences, University of California, Davis (http://tgrc.ucdavis.edu/). In addition, the tomato seeds of ‘Xin Jin Feng No. 1’ were purchased from the market (Xiankelian, Guangzhou, China), and this variety is commonly cultivated in the local area. The mutants *flc* (*flacca*), *sit* (*sitiens*), and *not* (*notabilis*) exhibit deficiencies in abscisic acid synthesis. The Micro‐Tom (MT) and Rheinlands Ruhm (RR) are considered wild types. Please refer to Table 1 for detailed information.

**TABLE 1:** ABA‐deficient mutant and wild‐type tomato cultivars used in this experiment.

Classification	Assoin number	Mutants/wild‐type	Background
*flc*	LA0673	Mutant	Rheinlands Ruhm
*sit*	LA0574	Mutant	Rheinlands Ruhm
*not*	LA4487	Mutant	Micro‐Tom
Micro‐Tom	LA3911	Wild‐type	

The symbiotic interaction between AM fungi and plants occurs primarily in the root system. To investigate how low pH in acidic soils affects AM fungal colonization, we adopted a sterile and well‐controlled experimental system. Given that roots are the primary site of symbiosis, the hairy root–AM fungi dual culture system allows us to precisely examine the molecular and physiological interactions under defined conditions.

Mutant and wild‐type seeds were subjected to sterilization followed by culturing in tissue culture bottles to obtain aseptic seedlings. The explants of these aseptic seedlings were then induced with *Agrobacterium rhizogenes* ACCC10060 (preserved at the Guangdong Provincial Microbial Culture Collection Center) for hairy root production, which were subsequently sterilized for future utilization.

### 2.1 The Fungi Material

The AM fungi *Ri*. DAOM197198 was acquired from Premier Tech in Canada and employed for the inoculation of tomato hairy roots in a dual culture system to propagate spores. The mature spores were utilized for the purpose of this study.

### 2.2 Medium

The culture medium utilized in this experiment is modified Strullu‐Romand (MSR) medium, following the methodology proposed by (Bécard and Fortin 1988). To account for the impact of high temperature and pressure sterilization on pH levels, a preliminary pH adjustment test was conducted to ensure attainment of the desired pH after high‐pressure sterilization. Various media with different pH values were prepared and subjected to high‐temperature and pressure sterilization, with their respective pH levels measured prior to solidification. The pre‐test results revealed that the medium at a pH of 4.5 remained virtually unchanged before and after sterilization; however, for the medium at a pH of 7.0, its post‐sterilization pH decreased to 6.3. Consequently, MSR media with initial pH values of 4.5 and 7.0 were prepared for subsequent high‐temperature and pressure sterilization (121°C for 15 min), poured into petri dishes within a laminar flow hood, cooled down, and solidified for future use.

## 3 Experimental Design

### 3.1 Dynamic Changes in Mycorrhizal Colonization and Abscisic Acid Content Under Rolonged Low pH Stress

The hairy roots of Xinjinfeng No. 1 tomato were used as the host material, and *RI*. DAOM197198 was employed as the AM fungi material. A three‐factor completely randomized experimental design was adopted in this study, with the following factors: the pH value of the medium (4.5 and 6.5), the inoculation status of AM fungi (non‐inoculated [−AM] and inoculated [+AM]), and the sampling time (4 weeks, 8 weeks, and 12 weeks). This design resulted in a total of 12 treatment combinations, with 7 biological replicates per combination.

Approximately 100 spores were evenly dispersed on each plate and incubated in a dark constant temperature incubator at 25°C for 5 days until spore germination occurred. Three approximately 3 cm long tomato hairy roots were placed from the edge to the center of each plate, maintaining a distance of about 1 cm between the roots and spores, and sealed with parafilm.

### 3.2 Determination of the Optimal ABA Concentration

Previous studies have demonstrated that the impact of ABA on AM fungal infection is concentration‐dependent, with different plant species exhibiting varying optimal concentrations. Therefore, we initially determined the optimum concentration of ABA in the culture medium. In this experiment, 9 cm plastic petri dishes were utilized to inoculate *Ri*. DAOM197198 spores using ‘Xinjinfeng No. 1’ tomato hairy roots as biological materials. Abscisic acid (ABA, Sigma‐Aldrich, A1049) was first dissolved in a trace amount of sodium hydroxide solution (10 μL, 1 M), then diluted to 1 mL with sterile water to prepare a 5 mM stock solution, which was stored at −20°C. Before use, the stock solution was diluted with distilled water to working solutions of 10^−4^ to 10^−8^ M. Different concentrations of ABA were added to MSR medium. The control group without ABA addition was included, with five replicates for each gradient. The inoculation of hairy roots and arbuscular mycorrhizal fungi was performed using the same method as previously described. After an incubation period of 8 weeks in a dark constant temperature incubator at 25°C, samples were collected.

### 3.3 Regulation of Arbuscule Development and Function by ABA Under Low pH Stress

The optimal concentration of abscisic acid (ABA) in MSR medium was determined to be 10^−7^ M, with no addition of ABA as the control. Different tomato ABA mutant hairy roots were utilized for the experiment, with wild‐type hairy roots serving as the control group. The mutant hairy roots were treated with ABA to observe its recovery effect. 15‐cm‐diameter Petri dishes were used to prepare MSR medium and establish two pH gradients (pH 4.5 and pH 6.5). A symbiotic association was formed between *Ri*. DAOM197198 spores and tomato hairy roots, while non‐inoculated AM fungi serving as the control. Except for increasing the number of hairy roots to 8, the inoculation of hairy roots with arbuscular mycorrhizal fungi was performed using the same method as previously described. Each treatment was performed in seven replicates, and plates were sealed with sealing film and incubated at a constant temperature of 25°C in darkness.

During sampling, transfer the culture medium along with the roots to a conical flask and add 200 mL of sodium citrate solution (10 mmol/L, pH 6.0). Incubate the flask on a shaker at 200 rpm for 30 min to dissolve the adhered MSR medium. The roots were then thoroughly washed with water, dried using absorbent paper, and weighed to determine their fresh weight. A carefully removing of old roots was performed while fine roots were cut into approximately 1 cm segments and homogeneously mixed. Some portions of the roots were stored in a refrigerator at 4°C for subsequent determination of mycorrhizal colonization, whereas other portions (0.2 g each) were preserved in the −80°C freezer for RNA extraction aimed at measuring gene expression levels relevant to this study.

### 3.4 ABA (Abscisic Acid, Sigma, A1049) Media With Different Concentrations

Prepared the 10^−2^ M ABA stock solution, which was then filtered and sterilized using the 0.22 μm inorganic polyethersulfone (PES) filter membrane. Subsequently, dilute the stock solution to 10^−4^ M and 10^−6^ M with sterile water. Finally, prepare MSR medium supplemented with ABA at concentrations ranging from 10^−4^ M to 10^−8^ M.

### 3.5 Mycorrhizal Colonization

The root fragments were stained with trypan blue according to the methods of Phillips and Hayman (1970), after which the quantification of mycorrhizal colonization was conducted according to the methods of Trouvelot, Kough, and Trouvelot et al. (1986) with the software MYCOCALC (http://www.dijon.inra.fr/mychintec/Mycocal‐prg/Download.html). Five parameters, namely F% (the colonization frequency), M% (the colonization intensity), m% (the relative colonization intensity), A% (the arbuscular abundance), and a% (the relative arbuscular abundance), were utilized to characterize the status of mycorrhizal colonization.

### 3.6 The Proportion of Arbuscular Development

To investigate the arbuscule development at different stages, the root fragments were double stained with WGA Alexa Fluor 488 and propidium iodide (PI) according to the methods described by Liu et al. (2020). The development of arbuscules was categorized into five distinct stages: hyphal invasion (I), initiation of arbuscule branching (II), arbuscule development (III), arbuscule maturation (IV), and arbuscule senescence (V).

### 3.7 Determination of ABA Content

ABA quantification was performed using HPLC with a UV detector (HPLC‐UV) (Comin, Suzhou, China) (Chen et al. 2020; Li et al. 2022). Approximately 0.2 g of root tissue stored at −80°C was ground using a cryogenic grinding apparatus. One milliliter of pre‐cooled methanol: acetic acid: water (12:3:5) solution was added, and the mixture was transferred to a 2 mL centrifuge tube for extraction at 4°C for 12 h. The sample was then centrifuged at 8000 × g for 10 min, and the supernatant was transferred to a new centrifuge tube. The precipitate was re‐extracted with 0.5 mL of the same extraction solution for 2 h and centrifuged again (8000 × g, 10 min). Another 0.5 mL of the extraction solution was added to the precipitate, which was sonicated in an ice bath for 20 min and centrifuged again (8000 × g, 10 min). All three supernatants were combined and evaporated under reduced pressure at 40°C until the organic phase was completely removed. The residue was extracted and decolorized with 0.5 mL of petroleum ether (repeated 3–5 times); the upper ether layer was discarded, and the lower layer was evaporated under reduced pressure at 40°C until dryness. The residue was dissolved in 0.5 mL of mobile phase, filtered through a needle filter into a sample vial with an inner liner for analysis.

The determination was performed using the Wufeng LC‐100 high‐performance liquid chromatograph equipped with an Anpel WP‐C18 chromatographic column (250 mm × 4.6 mm, 5 μm). The mobile phase was prepared by mixing methanol and acetic acid aqueous solution with a pH adjusted to 2.5 at a ratio of 30:70. The injection volume was set at 10 μL, the flow rate was 0.8 mL/min, the column temperature was controlled at 35°C, the total running time was 40 min, and the detection wavelength was set at 254 nm. Before formal sample addition, first balance the column with the mobile phase and start sample injection after the baseline was stable. The reagents used in the experiment include the hormone standard abscisic acid (ABA, purchased from Yuanye Biology), HPLC‐grade acetic acid (Aladdin), HPLC‐grade petroleum ether (Aladdin), HPLC‐grade methanol from Merck, and test water. Detailed validation data for this HPLC‐UV method confirm that peak identity and sensitivity were provided in Figures S1–S3 and Table S1.

### 3.8 qRT‐PCR Analysis of Selected Genes

Given the extensive number of samples, we carefully selected four biological replicates for each condition to ensure robust and well‐managed data analysis. RNA was extracted and reverse transcribed using the Plant RNA Kit (R6827, Omega Bio‐Tek, Guangzhou) and the TransScript One‐Step gDNA Removal Kit and cDNA Synthesis SuperMix (Beijing TransGen Biotech Co. Ltd.). qRT‐PCR was performed on the Roche LightCycler 480 II fluorescence quantitative PCR instrument. The 10 μL reaction mixture consisted of 5 μL iTaqTM Universal SYBR Green Supermix (2×), 0.5 μL of each forward and reverse primer (10 μmol/L), 1 μL cDNA template, and 3 μL RNase‐free and DNase‐free sterile deionized water. Thermal cycling conditions were as follows: initial denaturation at 94°C for 30 s, followed by 40 cycles of amplification (94°C for 5 s, 60°C for 30 s), and melting curve analysis from 65°C to 95°C (increasing by 0.5°C every 6 s). Each sample was analyzed in duplicate. Relative gene expression levels were calculated using the 2^−△△Ct^ method with actin as the internal reference gene. The sequences of the target genes and their corresponding primers were identical to those reported in our previous study (Liu et al. 2020). All primers were synthesized by the Guangzhou branch of Shanghai Sangon Biological Engineering Co. Ltd., and diluted to the working concentration according to the manufacturer's instructions prior to use.

### 3.9 Data Analysis

Due to contamination in certain samples during the experimental procedure, these samples were excluded from further analysis. For the uncontaminated samples, four to five data points demonstrating the highest repeatability were selected based on biomass measurements to ensure data accuracy. The data were subjected to multiple comparisons (Tukey HSD), Two‐way ANOVA analysis and independent‐sample *t*‐tests using IBM SPSS Statistics Version 21.0 (SPSS Inc., Chicago, IL). Multiple comparisons (Tukey's HSD and Dunnett's test) were consistently indicated using lowercase superscript letters to denote statistical groups; groups sharing the same letter were not significantly different. Significance tests (*t*‐tests) employed the asterisk notation system to represent significance levels (*p < 0.05, **p < 0.01, ***p < 0.001). For Two‐way ANOVA, significance was reported using *p*‐values. The figures were generated using Origin 8.5 software.

## 4 Result

### 4.1 Effects of Different Treatments on Root Biomass

**FIGURE 1:**
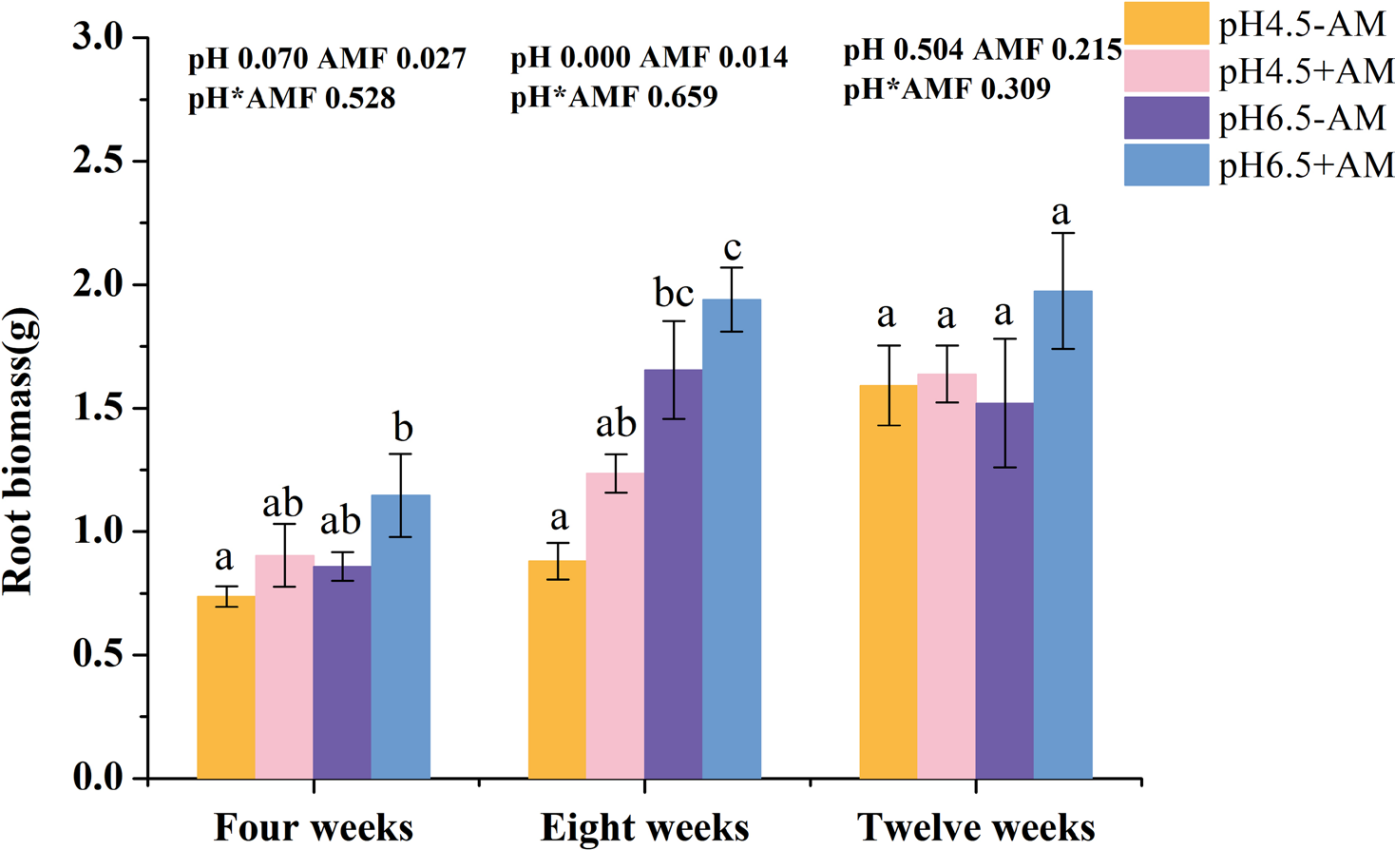
Root biomass of tomato hairy roots inoculated with/without AM fungi (+AM/−AM) under different pH treatments (pH 4.5/pH 6.5) at different culture times (4 weeks, 8 weeks, 12 weeks). All 4–7 means in each column followed by the same lowercase letter do not differ significantly by the Tukey HSD (*p* ≤ 0.05). The data presented at the top of the figure represents *p*‐values of the Two‐way ANOVA.

With the extension of culture time, root biomass exhibited a continuous increasing trend. Low pH tended to inhibit the increase in root biomass (Figure 1), whereas inoculation of AM fungi significantly enhanced root biomass at 4 weeks after inoculation (WAI) (*p* = 0.027, Figure 1). Both pH and AM fungal inoculation had significant effects on root biomass at 8 WAI. Specifically, low pH significantly decreased root biomass (Figure 1), while AM fungal inoculation markedly promoted its increase (Figure 1). However, the differences in root biomass among treatments were no longer significant at 12 WAI, potentially due to the roots entering the senescence stage and reaching the upper limit of nutrient utilization. Additionally, throughout the experiment, there was no significant interaction effect between pH level and AM fungal inoculation on root biomass (Figure 1).

### 4.2 Effect of Low pH on Mycorrhizal Colonization

**FIGURE 2:**
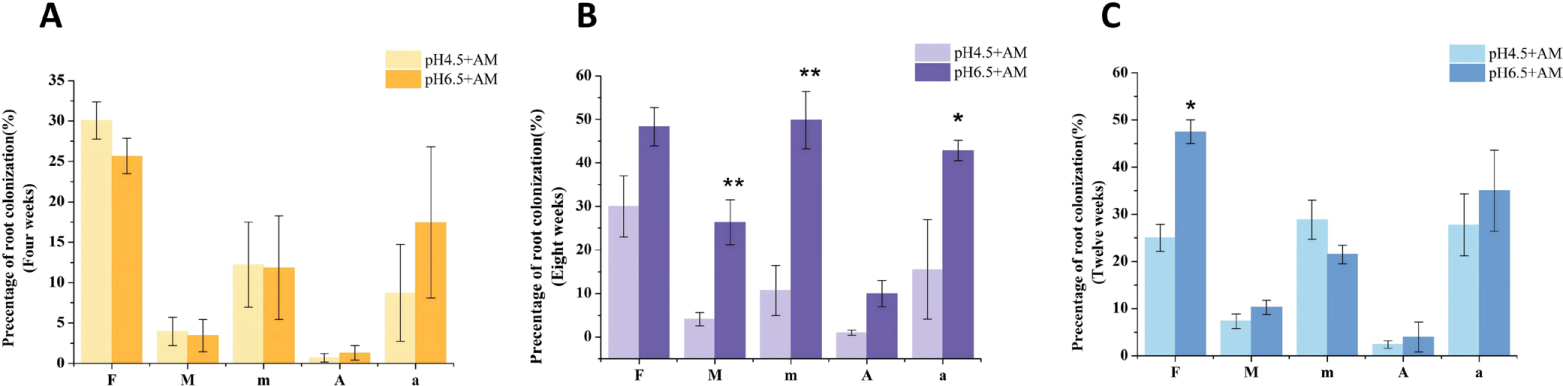
Mycorrhizal colonization of tomato hairy roots inoculated with AM fungi (+AM) under different pH treatments (pH 4.5/pH 6.5) at different culture times (4 weeks, 8 weeks, 12 weeks). (A) Mycorrhizal colonization at 4 weeks after inoculation (WAI) AM fungi; (B) Mycorrhizal colonization at 8 WAI; (C) Mycorrhizal colonization at 12 WAI. F%: The colonization frequency; M%: The colonization intensity; m%: The relative colonization intensity; A%: The arbuscular abundance; a%: The relative arbuscular abundance. Means (*n* = 4) are separated with independent‐samples *t*‐test (*p ≤ 0.05; **p ≤ 0.01).

With the extension of culture time, the mycorrhizal colonization gradually increases. There was no significant difference in the mycorrhizal colonization among the various treatments at 4 WAI (Figure 2). The low pH condition demonstrated an inhibitory effect on mycorrhizal colonization at 8 WAI (Figure 2). Specifically, both the mycorrhizal colonization intensity (M%) and the relative mycorrhizal colonization intensity (m%) were significantly higher in the pH 6.5 compared to the pH 4.5, suggesting that low pH levels may suppress the infection capacity of AM fungi. Apart from the mycorrhizal colonization frequency (F%) being significantly higher in the pH 6.5 treatment than in the pH 4.5 treatment, no significant differences were observed in other mycorrhizal colonization parameters at 12 WAI (Figure 2).

### 4.3 Effect of Low pH on the Development Dynamics of Arbuscules

**FIGURE 3:**
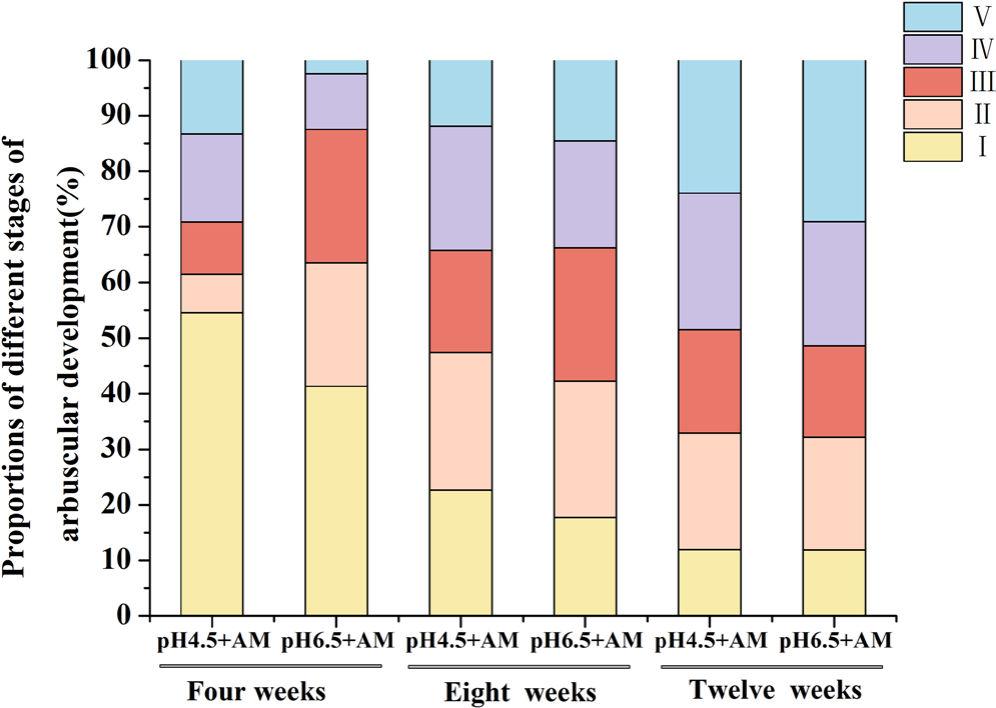
The proportion of arbuscular development of tomato hairy roots inoculated with AM fungi (+AM) under different pH treatments (pH 4.5/pH 6.5) at different culture times (4 weeks, 8 weeks, 12 weeks). The development of arbuscules was categorized into five distinct stages: Hyphal invasion (I), initiation of arbuscule branching (II), arbuscule development (III), arbuscule maturation (IV), and arbuscule senescence (V).

With the extension of culture time, arbuscules in the roots progressed from the juvenile stage to the mature and senescent stages. Specifically, the proportion of arbuscules in stage I gradually decreased, while the proportion of arbuscules in stage V gradually increased. At 4 WAI, the proportion of mycelium invasion (stage I) was the highest; at 8 WAI, the proportions of arbuscule development and maturation stages (II, III, IV) significantly increased; and at 12 WAI, the proportions of mature and senescent stages (IV, V) further increased (Figure 3). Additionally, the results demonstrated that low pH significantly delayed the developmental progression of arbuscules (Figure 3).

### 4.4 Effects of Low pH and Inoculated AM Fungi on ABA Content in Roots

**FIGURE 4:**
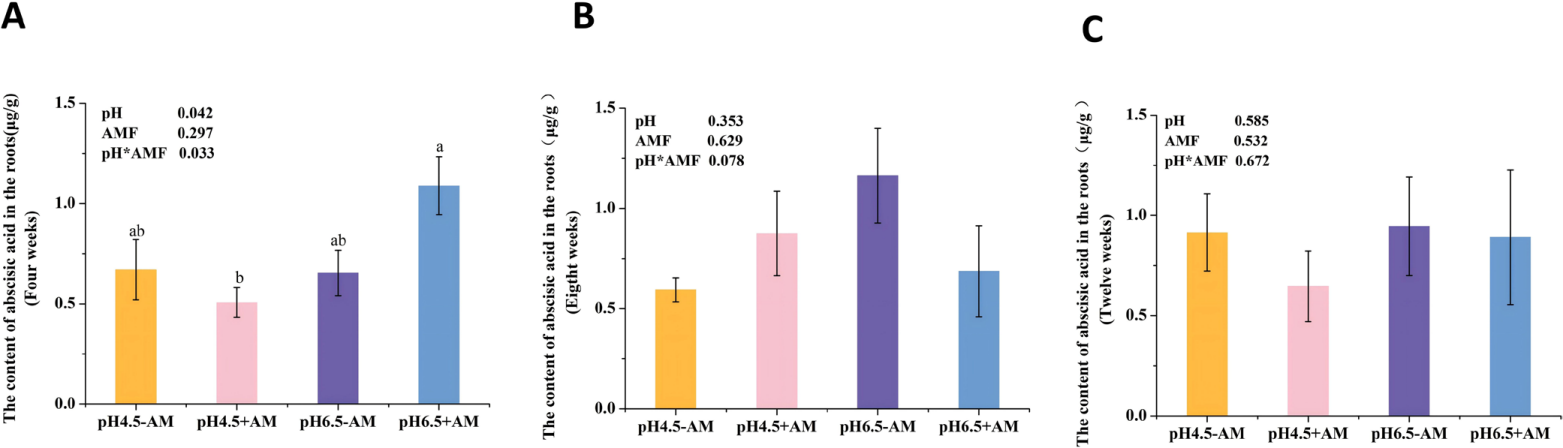
Abscisic acid content in the roots of tomato hairy roots inoculated with/without AM fungi (+AM/−AM) under different pH treatments (pH 4.5/pH 6.5) at different culture times (4 weeks, 8 weeks, 12 weeks). (A) The ABA content in the roots at 4 weeks after inoculation (WAI) with AM fungi; (B) The ABA content in the roots at 8 WAI; (C) The ABA content in the roots at 12 WAI. The averages in each column followed by the same lowercase letter do not differ significantly by the Tukey HSD (*p* ≤ 0.05). The data presented at the top of the figure represent *p*‐values of the Two‐way ANOVA.

The results indicated that during the 4th week, there were significant differences in ABA content in roots under various treatment conditions (Figure 4). Specifically, low pH significantly inhibited ABA accumulation in roots (*p* = 0.042), whereas inoculation with AM fungi did not significantly affect root ABA content. Additionally, the significant interaction effect between pH and AMF treatment was observed (*p* = 0.033) (Figure 4). However, during the 8th and 12th weeks, no significant differences in root ABA content were observed across treatments, and the interaction effect between pH and AMF treatment was also not significant (Figure 4).

### 4.5 The Optimal Concentration of ABA

**FIGURE 5:**
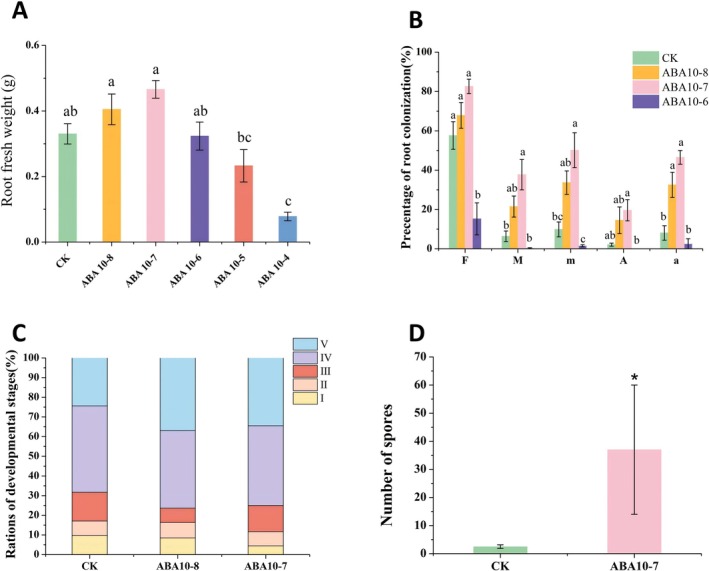
Optimal ABA concentration for promoting AM fungal infection and growth. (A) Effects of different ABA concentrations on root biomass of tomato; (B) Effects of different ABA concentrations on mycorrhizal colonization; (C) Effects of different ABA concentrations on arbuscular development; (D) Effects of ABA on spore production. ABA 10–8, ABA 10–7, ABA 10–6, ABA 10–5, ABA 10–4, respectively refers to the treatment with the exogenous application of 10^−8^ M, 10^−7^ M, 10^−6^ M, 10^−5^ M, 10^−4^ M abscisic acid. CK refers to the control treatment without the addition of exogenous ABA. The development of arbuscules was categorized into five distinct stages: Hyphal invasion (I), initiation of arbuscule branching (II), arbuscule development (III), arbuscule maturation (IV), and arbuscule senescence (V). The averages in each column followed by the same lowercase letter do not differ significantly by the Tukey HSD (*p* ≤ 0.05). Means are separated with independent‐samples *t*‐test (*p ≤ 0.05).

As shown in Figure 4A, when the ABA concentration was 10^−4^ M and 10^−5^ M, the biomass was significantly lower than that of the control group. In contrast, the biomass was higher than that of the control at concentrations of 10^−7^ M and 10^−8^ M. Notably, except for the 10^−4^ M treatment group (where the roots failed to grow entirely), there were no significant differences between the other groups and the control group (Figure 5A). Due to severely stunted root development in the 10^−4^ M and 10^−5^ M treatments, which prevented normal growth, the mycorrhizal colonization was nearly undetectable. Consequently, colonization data for these groups was excluded from the analysis. The mycorrhizal colonization treated with 10^−7^ M ABA was the highest among the treatments of 10^−6^ M, 10^−7^ M, and 10^−8^ M. Specifically, the colonization intensity (M%), relative colonization intensity (m%), and relative arbuscular abundance (A%) in 10^−7^ M ABA were significantly greater than those in control (Figure 5B). Collectively, these results indicate that the 10^−7^ M ABA treatment was the most effective in promoting mycorrhizal colonization (Figure 5B).

No arbuscular structures were observed in the mycorrhizae treated with ABA at 10^−4^ M, 10^−5^ M, and 10^−6^ M. The proportion of senescent arbuscules (stage V) in the ABA 10^−7^ M and 10^−8^ M treatments was significantly higher than that of the control, suggesting that ABA accelerated the developmental process of arbuscules (Figure 5C). Further analysis revealed that under the ABA 10^−7^ M treatment, the proportion of robust and functionally superior arbuscules was higher, indicating a more favorable developmental status compared to the ABA 10^−8^ M treatment. Additionally, regarding sporulation, the ABA 10^−7^ M treatment resulted in significantly higher numbers than the control (Figure 5D).

Considering the comprehensive results of biomass, mycorrhizal colonization, arbuscule developmental proportions, and sporulation, 10^−7^ M was ultimately determined to be the optimal ABA concentration for promoting the infection of AM fungi to roots.

### 4.6 Effects of Different Treatments on Root Biomass

There are no significant differences in the biomass of wild‐type MT across different pH levels, abscisic acid (ABA) treatments, or arbuscular mycorrhizal fungi (AMF) inoculations. For the abscisic acid‐deficient mutant *not*, neither pH levels nor AMF significantly affect its biomass. In the presence of abscisic acid (+ABA), the biomass was significantly greater than in its absence (−ABA) (Table 2).

**TABLE 2:** Influence of low pH, AM fungi inoculation and exogenous ABA on root biomass.

Genotype	Root biomass (g/plate)	
	pH 4.5	pH 6.5
*not*	2.86 ± 0.10	2.8 ± 0.10
MT	2.73 ± 0.05	2.62 ± 0.16
	**−AMF**	**+AMF**
*not*	2.86 ± 0.14	2.79 ± 0.06
MT	2.82 ± 0.08	2.52 ± 0.14
	**−ABA**	**+ABA**
*not*	2.46 ± 0.06	3.15 ± 0.06***
MT	2.68 ± 0.04	2.67 ± 0.16
**Two‐way ANOVA (*p* value)**		
pH	0.006	
ABA	0.449	
pH*ABA	0.825	

*Note:* Independent‐samples *t*‐test and Two‐way ANOVA were performed on biomass treated with different pH, ABA, and AMF. Data are presented as average ± standard error. “***” indicates significant difference between ABA treatments at *p* ≤ 0.001. PH4.5 and pH 6.5 respectively represent pH 4.5 and pH 6.5 treatments; +ABA and −ABA respectively denote the treatments with and without exogenous ABA addition; +AM and −AM indicates the treatment with and without AM fungi inoculation.

Abbreviations: MT, Micro‐Tom; *Not*, ABA‐deficient mutant.

### 4.7 Regulatory Pattern of Abscisic Acid on AM Fungal Infection Under Low pH Conditions

**FIGURE 6:**
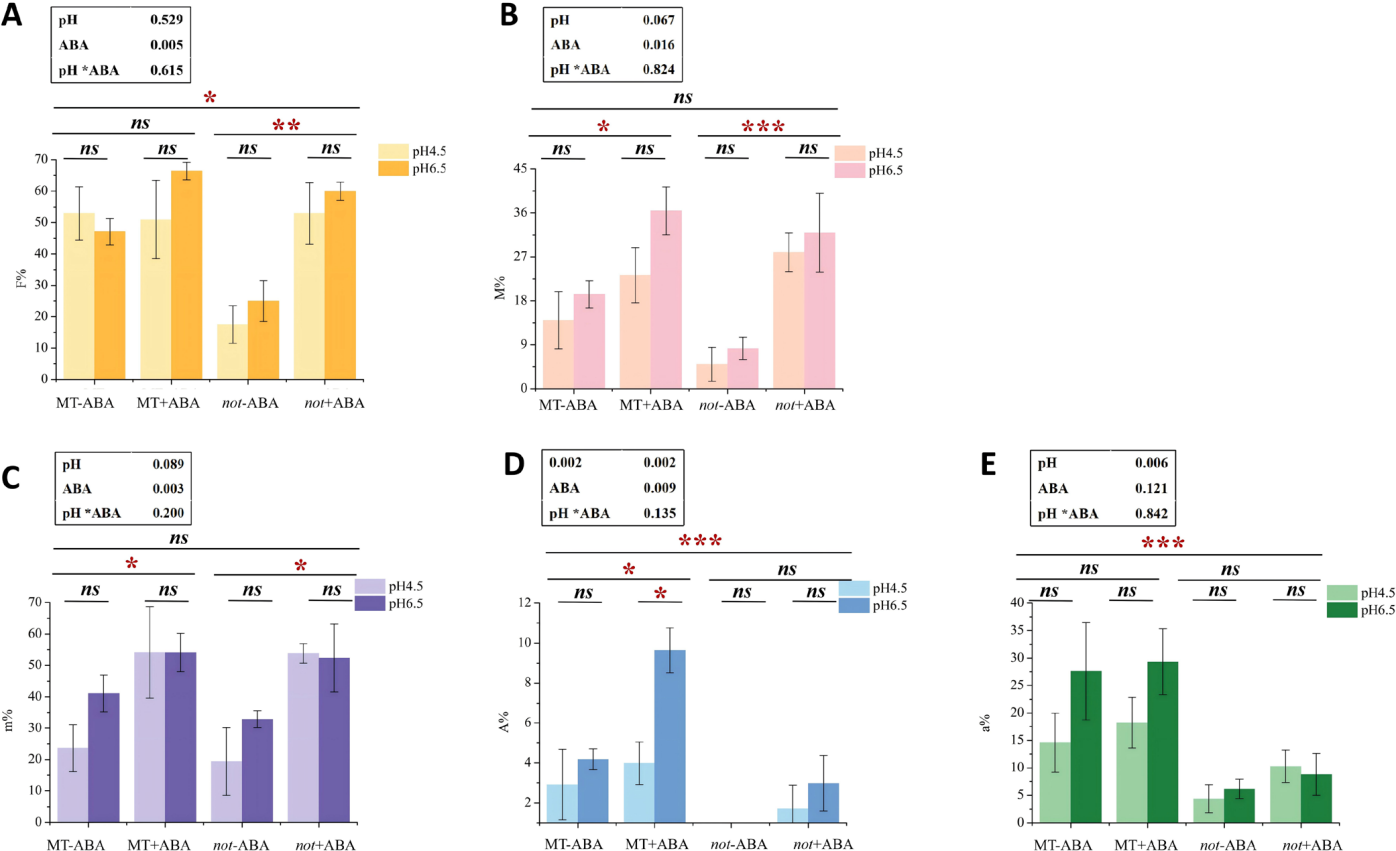
Mycorrhizal colonization of wild‐type (MT) and ABA‐deficient mutant (*not*) tomato hairy roots under different pH and ABA treatments. (A) the colonization frequency F%; (B) The colonization intensity M%; (C) The relative colonization intensity m%; (D) The arbuscular abundance A%; (E) The relative arbuscular abundance a%. “*”, “**”, “***” indicate that the mycorrhizal colonization between different treaments is significant difference (*p < 0.05, **p < 0.005, ***p < 0.001), and “ns” indicates that there is no significant difference between different treatments. The data presented in the black box above the figure represents *p*‐values of the Two‐way ANOVA.

Low pH significantly decreased the arbuscular abundance (A%) in wild‐type MT (Figure 6D), but did not significantly affect other colonization parameters. In the mutant, F%, A%, and a% were all significantly lower than those in the wild type (Figure 6A,D,E). Exogenous application of ABA significantly enhanced the infection intensity (M%, Figure 6B), relative infection intensity (m%, Figure 6C), and arbuscular abundance (A%, Figure 6D) in wild‐type. Moreover, exogenous ABA also significantly increased F%, M%, and m% in the mutant (Figure 6A–C). These results suggest that exogenous ABA partially restored the mycorrhizal colonization. Two‐way ANOVA analysis further revealed that ABA treatment had a significant effect on F%, M%, m%, and A%, while pH significantly influenced A% and a% (Figure 6). Overall, the mycorrhizal colonization in the abscisic acid‐deficient mutant was generally lower than that in the wild type, indicating that ABA deficiency inhibits mycorrhizal colonization, whereas ABA supplementation promotes it.

**FIGURE 7:**
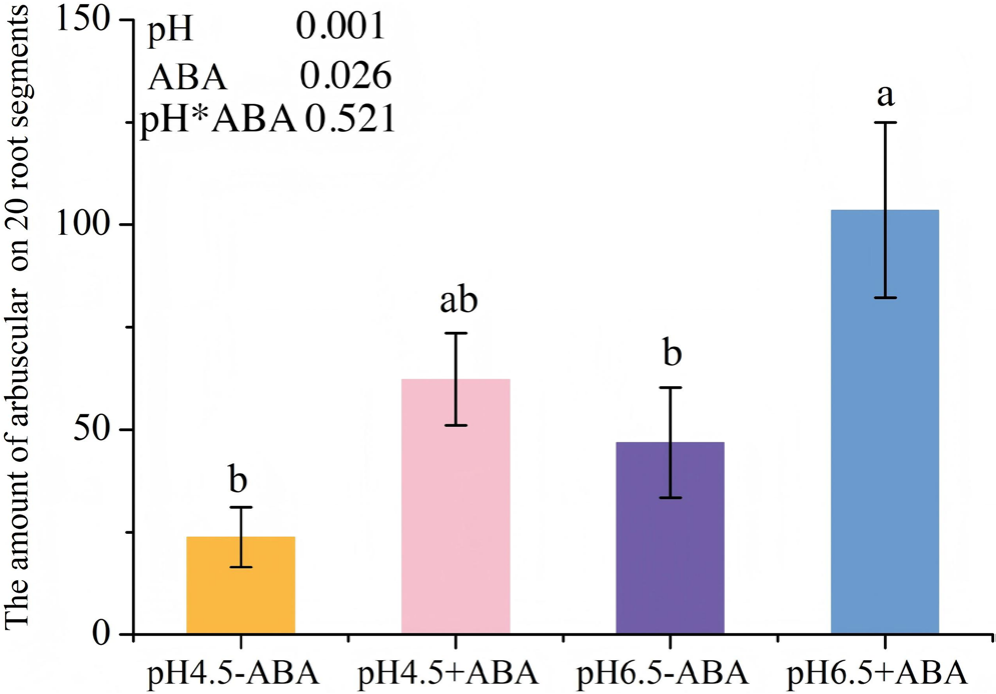
Under different pH and abscisic acid (ABA) treatments, 20 randomly selected wild‐type (MT) tomato hairy roots stained with trypan blue were used to count the total number of arbuscules. −4.5 and −6.5 respectively represent pH 4.5 and pH 6.5 treatments; +ABA and −ABA respectively denote the treatments with and without exogenous ABA addition. The averages in each column followed by the same lowercase letter do not differ significantly by the Tukey HSD (*p* ≤ 0.05). The data presented at the top of the figure represents *p*‐values of the Two‐way ANOVA.

Furthermore, analysis was performed on the number of arbuscules in the roots. The results demonstrated that under ABA treatment, the number of arbuscules significantly increased (*p* = 0.001) (Figure 7). In contrast, under low pH conditions, the formation of arbuscules was markedly inhibited (*p* = 0.026) (Figure 7). Moreover, there was no significant interaction effect between ABA and pH on arbuscule abundance (Figure 7).

### 4.8 Effects of Abscisic Acid on the Development Process of Arbuscules Under Low pH Conditions

Owing to the large number of experimental treatments, the data were transformed to ensure clarity and comparability. According to our previous study (Liu et al. 2020), the five stages of arbuscular development (I, II, III, IV, V) were quantified as numerical values 1, 2, 3, 4, and 5, respectively. These values were used to evaluate changes in arbuscular development status under different treatment conditions. Specifically, the numerical value closer to 5 indicates that the arbuscule is closer to the senescence state. The results demonstrated that under low pH conditions, the arbuscular development status in roots of different genotypes tended to remain at the juvenile stage, particularly reaching a significant level in the mutant *sit* and not roots (Table 3). In contrast, arbuscules under normal pH treatment exhibited the mature state. However, exogenous ABA treatment did not significantly affect arbuscular development (Table 3), suggesting that the addition of exogenous ABA did not alter the developmental process of arbuscules. Furthermore, two‐way ANOVA analysis revealed that low pH significantly inhibited arbuscular development, while ABA had no significant effect on arbuscular development, and there was no interaction effect between pH and ABA (Table 3).

**TABLE 3:** Influence of low pH and exogenous ABA on the development of arbuscules.

Genotype	Development of arbusclue	
	pH 4.5	pH 6.5
*sit*	2.67 ± 0.17	3.40 ± 0.09**
*flc*	2.91 ± 0.30	3.37 ± 0.14
*not*	2.59 ± 0.21	3.34 ± 0.14*
MT	3.12 ± 0.25	3.42 ± 0.16
	−ABA	+ABA
*sit*	3.26 ± 0.17	2.96 ± 0.23
*flc*	3.11 ± 0.38	3.06 ± 0.22
*not*	3.08 ± 0.29	3.10 ± 0.16
MT	3.45 ± 0.16	3.08 ± 0.24

*Note:* Independent‐samples *t*‐test were performed on the development of arbusclues treated with different pH and ABA. Data are presented as average ± standard error. “**” and “*” indicates significant difference between differern treatments at *p* ≤ 0.005 and *p* ≤ 0.05. PH4.5 and pH 6.5 respectively represent pH 4.5 and pH 6.5 treatments; +ABA and −ABA respectively denote the treatments with and without exogenous ABA addition.

### 4.9 The Regulatory Pattern of Abscisic Acid on the Function of AM Fungi Under Low pH

**FIGURE 8:**
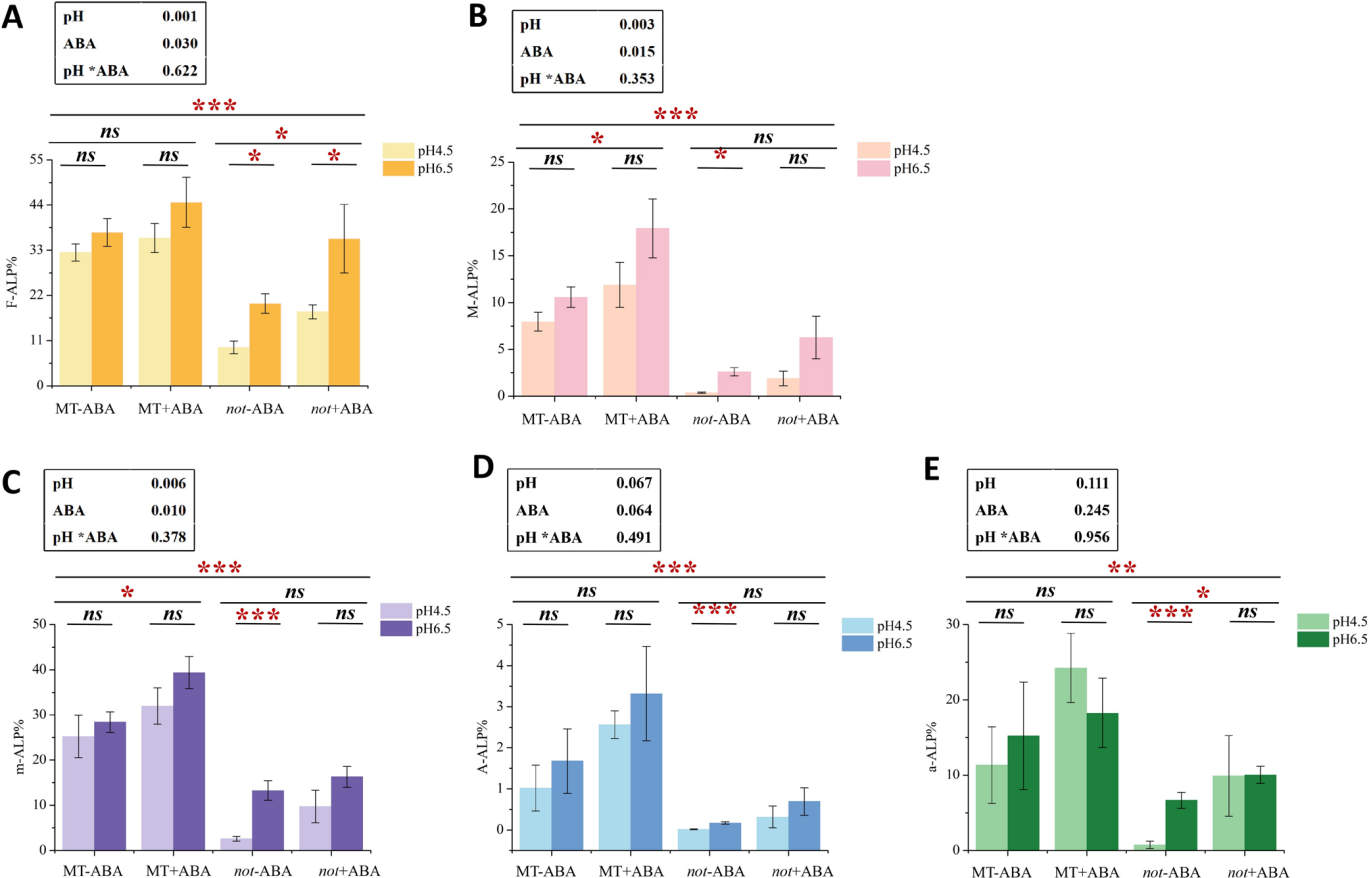
Alkaline phosphatase activity in wild‐type (MT) and ABA‐deficient mutant (*not*) tomato hairy roots under different pH and ABA treatments. (A) F‐ALP%, Mycorrhizal frequency with ALP activity in the roots; (B) M‐ALP%, Mycorrhizal intensity with ALP activity in the roots; (C) M‐ALP%, Mycorrhizal intensity with ALP activity in colonized roots; (D) A‐ALP%, Arbuscule abundance with ALP activity in the roots; (E) A‐ALP%, Arbuscule abundance with ALP activity in colonized roots. “*”, “**”, “***” indicate that the alkaline phosphatase activity between different treaments is significant difference (*p < 0.05, **p < 0.01,***p < 0.001), and “ns” indicates that there is no significant difference between different treatments. The data presented in the black box above the figure represents *p*‐values of the Two‐way ANOVA.

The alkaline phosphatase (ALP) activity under different treatments was assessed. The results demonstrated that low pH did not significantly affect the ALP activity in wild‐type roots but significantly inhibited the F%, M%, m%, A%, and a% in the mutant *not* roots (Figure 8A–E). When ABA was applied to the mutant *not* roots, low pH significantly inhibited F% (Figure 8A) while having no significant effect on other ALP parameters. These results suggest that exogenous ABA can partially alleviate the inhibition of ALP activity caused by low pH.

The application of ABA significantly enhanced the infection intensity (M%, Figure 8B) and relative infection intensity (m%, Figure 8C) of ALP activity in wild‐type roots, as well as significantly promoted F% and a% in the mutant *not* (Figure 8A,E). Compared with the wild type, all ALP‐related parameters in the ABA‐deficient mutant were significantly lower than those in the wild type, suggesting that the defect in root ABA synthesis inhibited the function of AM fungi (Figure 8).

Furthermore, Two‐way ANOVA analysis revealed that the application of ABA enhanced the function of AM fungi, with significant effects observed on F% (*p* = 0.030), M% (*p* = 0.015), and m% (*p* = 0.010) (Figure 8A–C). In contrast, low pH significantly suppressed the function of AM fungi, with notable impacts on F% (*p* = 0.001), M% (*p* = 0.003), and m% (*p* = 0.006) (Figure 8A–C).

The results of quantitative analysis of the relative expression levels of the phosphate transporter genes *SlPTs* indicated that low pH significantly inhibited the expression of the *SlPT1* and *SlPT5* genes, while abscisic acid (ABA) treatment had no significant effect on the overall relative expression level of the *SlPT* gene family. In addition, inoculation of AM fungi significantly increased the expression levels of the *SlPT1*, *SlPT2*, and *SlPT5* genes (Table 4).

**TABLE 4:** Effects of AM fungi inoculation and exogenous ABA on the relative expression levels of *SlPTs* genes in roots under low pH conditions.

Genes	Relative expression level	
	pH 4.5	pH 6.5
*SlPT1*	1.14 ± 0.10	1.48 ± 0.09*
*SlPT2*	2.88 ± 0.53	3.42 ± 0.80
*SlPT3*	1.78 ± 0.16	1.73 ± 0.16
*SlPT5*	0.95 ± 0.19	2.31 ± 0.51*
	**−ABA**	**+ABA**
*SlPT1*	1.35 ± 0.11	1.27 ± 0.08
*SlPT2*	3.27 ± 0.53	3.03 ± 0.79
*SlPT3*	1.81 ± 0.17	1.71 ± 0.14
*SlPT5*	1.69 ± 0.36	1.57 ± 0.45
	**‐AMF**	**+AMF**
*SlPT1*	1.00 ± 0.05	1.62 ± 0.10***
*SlPT2*	2.27 ± 0.31	4.05 ± 0.89
*SlPT3*	1.31 ± 0.11	2.21 ± 0.16***
*SlPT5*	0.59 ± 0.08	2.67 ± 0.5***

*Note:* “***” and “*” indicates significant difference between different treatments at *p* ≤ 0.001 and *p* ≤ 0.05. PH4.5 and pH 6.5 respectively represent pH 4.5 and pH 6.5 treatments; +ABA and −ABA respectively denote the treatments with and without exogenous ABA addition; +AM and −AM indicates the treatment with and without AM fungi inoculation.

### 4.10 Effect of Abscisic Acid on *EXO70*s and Lipid Biosynthesis/Transport‐Related Gene Expression Under Low pH

The expression patterns of the *EXO70s* gene family in roots under low pH and exogenous ABA treatments were analyzed. The results demonstrated that low pH significantly suppressed the expression of the *EXO70A1‐like* (LOC101253481) gene, whereas it had no significant effect on the expression levels of the *EXO70A1‐like* (LOC101261477) and *EXO70B1* genes (Table 5). Moreover, compared with the wild type, the expression of the *EXO70A1‐like* (LOC101253481) gene was markedly reduced in the *not*‐mutant, while no significant differences were observed in the expression levels of the *EXO70A1‐like* (LOC101261477) and *EXO70B1* genes between the *not*‐mutant and its wild type (Table 6).

**TABLE 5:** Effects of exogenous ABA on the relative expression levels of *EXO70s* genes in infected roots under low pH conditions.

Gene	Relative expression level	
	−ABA	+ABA
*EXO70A1‐like* (LOC101253481)	1.05 ± 0.16	1.07 ± 0.16
*EXO70A1‐like* (LOC101261477)	1.13 ± 0.09	1.25 ± 0.17
*EXO70B1*	0.92 ± 0.10	0.92 ± 0.12
	**pH 4.5**	**pH 6.5**
*EXO70A1‐like* (LOC101253481)	0.75 ± 0.11	1.37 ± 0.10***
*EXO70A1‐like* (LOC101261477)	1.17 ± 0.07	1.23 ± 0.18
*EXO70B1*	0.90 ± 0.19	0.94 ± 0.09

*Note:* “***” indicates significant difference between different treatments at *p* ≤ 0.001. PH4.5 and pH 6.5 respectively represent pH 4.5 and pH 6.5 treatments; +ABA and −ABA respectively denote the treatments with and without exogenous ABA addition.

**TABLE 6:** The relative expression levels of *EXO70s* genes in different genetypes roots.

Genetypes	*EXO70A1‐like* (LOC101253481)	*EXO70A1‐like* (LOC101261477)	*EXO70B1*
*not*	0.31 ± 0.06	1.03 ± 0.04	0.89 ± 0.11
MT	1.63 ± 0.30***	0.98 ± 0.06	1.00 ± 0.09

*Note:* “***” indicate significant difference between different treatments at *p* ≤ 0.001. PH4.5 and pH 6.5 respectively represent pH 4.5 and pH 6.5 treatments; +ABA and −ABA respectively denote the treatments with and without exogenous ABA addition.

Abbreviations: MT, Micro‐Tom; *Not*, ABA‐deficient mutant.

**FIGURE 9:**
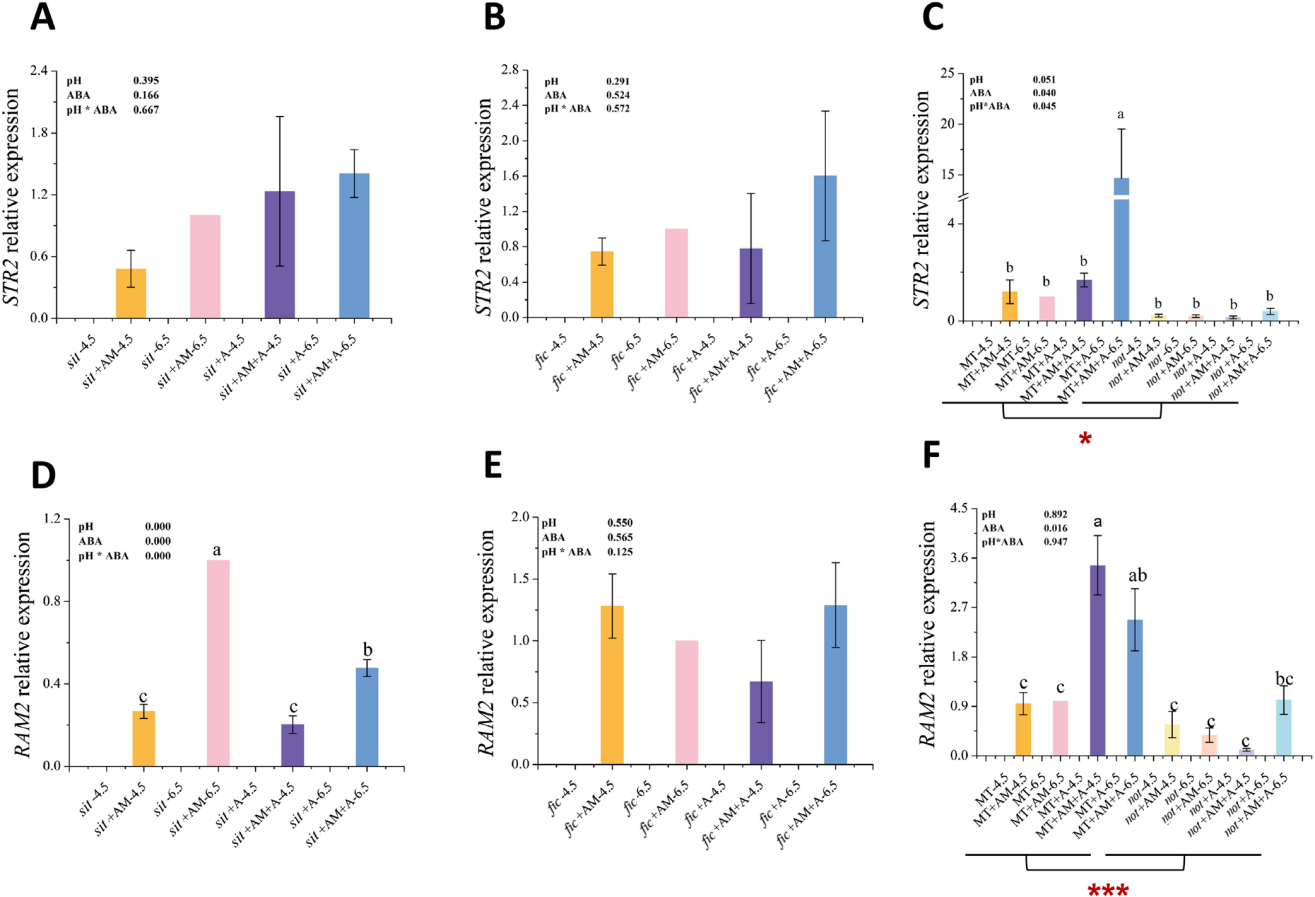
Effects of different treatments on the expression of genes related to fatty acid synthesis and transport. *flc: Flacca*, *sit*: *Sitiens*; *not*: *Notabilis*; MT: Micro‐Tom Take the wild type as an example to explain the meanings of different treatments: MT + A‐6.5: The roots of wild‐type MT treated with exogenous ABA addition, without inoculation of AM fungi, and at pH 6.5: MT + AM+A‐6.5: The rootss of wild‐type MT treated with exogenous ABA addition, inoculated with AM fungi, and at pH 6.5. The averages in each column followed by the same lowercase letter do not differ significantly by the Tukey HSD (*p* ≤ 0.05). “*” and “***” indicate significant difference between different treatments at *p* ≤ 0.05 and *p* ≤ 0.001. The data presented at the top of the figure represents *p*‐values of the Two‐way ANOVA.

The relative expression levels of the *STR2* and *RAM2* were analyzed. The results revealed that these genes were undetectable under non‐inoculated conditions, yet showed specific upregulation following inoculation. Further investigation revealed that ABA application significantly enhanced the expression of both *STR2* and *RAM2* in the *not*‐mutant and its wild‐type MT (*p* = 0.040 and *p* = 0.016, respectively). Conversely, in the ABA‐deficient *not* mutant roots, the expression levels of *STR2* and *RAM2* were markedly suppressed compared to the wild type (*p* = 0.003 and *p* = 0.001, respectively) (Figure 9).

### 4.11 The Effects of Exogenous ABA on Endogenous ABA Content in Mycorrhizae Under Low pH

**FIGURE 10:**
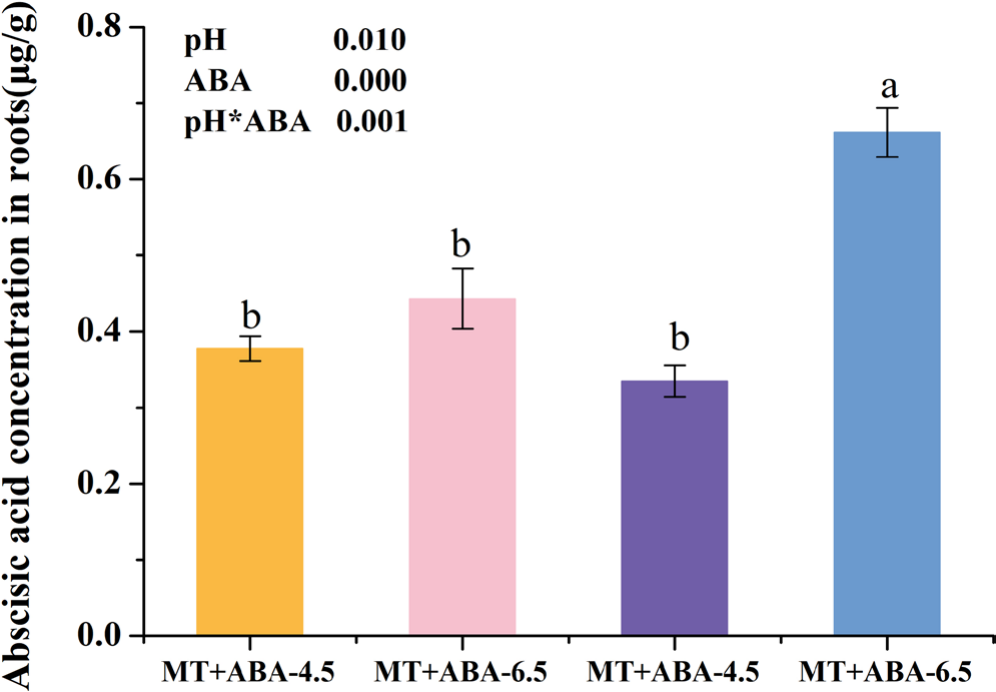
Effects of different pH and ABA treatments on ABA content in wild‐type (MT) tomato hairy roots inoculated with AM fungi. −4.5 and −6.5 respectively represent pH 4.5 and pH 6.5 treatments; +ABA and −ABA respectively denote the treatments with and without exogenous ABA addition. The averages in each column followed by the same lowercase letter do not differ significantly by the Tukey HSD (*p* ≤ 0.05). The data presented at the top of the figure represent *p*‐values of the Two‐way ANOVA.

As the root biomass of the mutant was significantly lower than that of the wild type, we focused our analysis on the ABA content in the roots of the wild type only. The results indicated that exogenous ABA treatment significantly increased the ABA content in the roots of the wild type (*p* = 0.000). Additionally, in MT mycorrhizae, low pH significantly suppressed ABA content (*p* = 0.010), while exogenous ABA treatment significantly increased ABA content (Figure 10). Notably, a significant interaction effect of pH and exogenous ABA on ABA content was detected in MT (*p* = 0.001) (Figure 10).

## 5 Discussion

### 5.1 There Are Significant Differences in the Sensitivity of Tomato Hairy Roots of Various Genotypes to Low pH

AM fungi significantly increased root biomass at 4WAI and 8WAI (Figure 1). Thses results were consistent with prior research findings (Zhang et al. 2025; Wang et al. 2025; Xiao et al. 2025; Bryant and Bever 2025), which collectively demonstrate that AM fungi can significantly enhance plant biomass. However, neither pH nor AM fungi had a significant effect on root biomass at 12 WAI. It is probable that the absorption and utilization of nutrients by the roots were constrained in the Petri dish at 12 WAI, and the roots concurrently began transitioning into the aging phase. Consequently, the impact of environmental treatments on root biomass became relatively restricted (Figure 1). In addition, the low pH significantly inhibited the growth of root biomass at 8 WAI, which can be attributed to the inhibitory effect of acidic conditions on root development (Lv et al. 2024; Du et al. 2024).

Under different pH conditions, significant differences in biomass and mycorrhizal colonization were observed at 8 WAI (Figure 2). This suggests that mycorrhizae exhibited the strongest response to pH changes at this specific time point. Low pH and inoculation with AM fungi had no significant effect on the biomass of wild‐type tomato hairy roots. The roots of the locally cultivated variety (Xin Jinfeng No. 1) were not significantly influenced by pH at 4 and 12 WAI, but exhibited a significant response specifically at the 8 WAI. This implies that tomato hairy roots of different genotypes may exhibit differential responses to pH changes at distinct time points. In the wild‐type (MT) and mutant (*not*), it is possible that the roots had not yet reached a critical response threshold by the 8th week of culture or had already passed through the period of heightened sensitivity to pH.

Apart from using tomato hairy roots of different genotypes, all other experimental conditions remained consistent. Therefore, it can be inferred that there are significant differences in the sensitivity of tomato hairy roots of various genotypes to low pH. Wang et al. (2017) used the hairy roots of ‘Xin Jinfeng No. 1’ as plant material and discovered that low pH substantially inhibited root biomass growth, which is consistent with our results. Additionally, Yang et al. (2018) employed Xuegan ( *Citrus sinensis* ) and Suanyou ( *Citrus grandis* ) as plant material and conducted planting experiments in nutrient solutions of differing pH levels. The results indicated that treatments at pH 6 and pH 3 had no significant impact on root biomass for both plants, which is consistent with the results of the wild‐type (MT) and mutant (*not*). Despite low environmental pH, roots can adapt to external pH stress through osmotic pressure regulation. Ultimately, the physiological function of roots is determined by the intracellular pH level. Due to variations in osmotic pressure regulation capabilities among tomato hairy roots of different genotypes, they exhibit distinct response mechanisms under acid stress conditions.

It is worth that ‘New Jinfeng No. 1’, as the widely cultivated commercial variety in the market, may exhibit greater adaptability to normal or nutrient‐rich growth conditions. Following long‐term domestication under cultivation conditions, it demonstrates higher yields and improved quality. Simultaneously, due to its prolonged exposure to a stable and favorable environment, its capacity to withstand environmental stress has diminished, making it more sensitive to environmental changes. Most wild‐type and mutant plant materials are primarily utilized for germplasm resource conservation or scientific research. These materials have not been subject to long‐term cultivation or domestication, thus retaining a stronger capacity for environmental adaptation.

### 5.2 Low pH Inhibits Mycorrhizal Colonization and Arbuscule Development

The results demonstrated that low pH significantly inhibited the infection process of AM fungi on the roots. Low pH significantly reduced the mycorrhizal colonization frequency (F%) at 4 WAI and significantly suppressed the mycorrhizal colonization intensity (M%) and relative infection intensity (m%) at 8 WAI (Figure 2). Through the dynamic observation of arbuscular development under different treatment conditions, it was found that the arbuscules developmental stage gradually transformed from the arbuscules to the mature and senescent stage as the culture time increased (Figure 3). However, low pH significantly delayed this development process (Figure 3). Additionally, the results showed that low pH significantly reduced the abundance of arbuscules in wild‐type MT, but had no significant effect on other mycorrhizal colonization parameters (Figure 6 and Figure S4). This indicated that arbuscules were more sensitive to the low pH environment (Feng et al. 2020; Wang et al. 2017). Furthermore, in the roots of the mutant, low pH significantly inhibited m%, A%, and a%, suggesting that the infection process in ABA‐deficient roots is more sensitive to pH changes. This could potentially result from the combined effects of ABA deficiency and low pH stress (Figure 6).

Previous studies have demonstrated that low pH environment imposes significant stress on the growth and development of AM fungi, markedly inhibiting hyphal expansion (Liu et al. 2020; Feng et al. 2020). This inhibition further hinders the formation and development of arbuscules, thereby reducing the infection efficiency and functional performance of AM fungi (Liu et al. 2020; Feng et al. 2020; Ahmed et al. 2025). Additionally, low pH may indirectly restrict the colonization and development of AM fungi by suppressing plant root growth, particularly lateral roots (Feng et al. 2025). Notably, while acidic soil can impede the normal development of arbuscules, normal arbuscular structures can still be observed under low pH treatment. The impaired development of arbuscules in acidic soils might result from increased concentrations of toxic heavy metal ions and reduced soil quality due to acidification (Pedron et al. 2009; Wang et al. 2025; Łukaszek‐Chmielewska et al. 2025). Low pH not only significantly reduces arbuscular abundance but also delays their developmental progression. Under low pH conditions, arbuscules tend to remain at the juvenile stage, with a higher proportion of functionally weaker primary structures, whereas the truly functional components are the highly branched fine terminal structures, specifically stages III and IV (Liu et al. 2020). Moreover, a low pH significantly decreases the number of arbuscules (Figure 7) and markedly suppresses the expression of the key gene *EXO70A1‐like* (LOC101253481), which is critical for arbuscular development (Table 5).

Arbuscules are the central sites for material exchange in mycorrhizal symbionts (Walder and van der Heijden 2015; Luginbuehl and Oldroyd 2017; Wang et al. 2017; Wipf et al. 2019). Host plants and AM fungi utilize this structure to achieve efficient exchange of substances and signals (Díaz et al. 2025). Specifically, AM fungi transport nutrients and water absorbed via extraradical hyphae to the host plants through arbuscules, while simultaneously acquiring carbon sources produced by the host plant (Bell et al. 2024; Magkourilou et al. 2025). As a key structure, arbuscules play an indispensable role in promoting the growth of both AM fungi and host plants. Research by Feng et al. (2020) further demonstrated that, compared with other infection indicators, arbuscular abundance is significantly positively correlated with plant biomass and markedly increases phosphorus concentration in the aboveground parts (Feng et al. 2020). Thus, arbuscules represent the core functional manifestation of the AM fungal symbiotic system, and their development status and quantity directly determine the physiological performance of AM fungi. However, low pH significantly suppresses mycorrhizal colonization and arbuscular development (Feng et al. 2020; Liu et al. 2020). Further studies have revealed that under low pH conditions, the proportion of functionally active alkaline phosphatase decreases substantially (Figure 8), and the expression levels of key mycorrhizal function genes, such as *SlPT1* and *SlPT5* from the *SlPTs* family, also decrease significantly (Table 4). In summary, low pH primarily weakens mycorrhizal function by inhibiting arbuscular development.

### 5.3 The Appropriate Concentration of Abscisic Acid (ABA) Can Promote the Mycorrhizal Colonization and Functional of AM Fungi

Previous studies have shown that the effect of abscisic acid (ABA) on mycorrhizal colonization is concentration‐dependent (Herrera‐Medina et al. 2007; Charpentier et al. 2014). To determine the optimal ABA concentration for promoting mycorrhizal colonization, this study conducted experiments using media supplemented with varying concentrations of ABA. The findings revealed that high concentrations of ABA significantly inhibited mycorrhizal colonization, whereas appropriate concentrations of ABA significantly enhanced the colonization. Furthermore, the appropriate concentration of ABA could increase the activity of alkaline phosphatase (Figure 8 and Figure S5), but had no significant impact on arbuscule development. Compared with the wild type, the expression level of the gene *EXO70A1‐like* (LOC101253481), which is associated with arbuscule development, was significantly decreased in the *not* mutant (Table 6). These results indicate that ABA can restore the arbuscular development process in ABA‐deficient mutants. Extensive studies have reported that ABA can promote mycorrhizal colonization, and the results of this study are consistent with these prior conclusions (Charpentier et al. 2014; Jing et al. 2025; Kandalgaonkar and Barvkar 2025).

In this study, the application of ABA had no significant effect on the developmental process of arbuscules. This suggests that ABA can enhance mycorrhizal colonization and increase the proportion of arbuscules, while it does not accelerate the senescence or collapse of arbuscules. Moreover, although ABA significantly increased alkaline phosphatase activity in the roots (Figure 8), it did not significantly up‐regulate the expression of phosphate transporter‐related genes (*SlPT*s) at the transcriptional level (Table 4). This phenomenon may be attributed to the high nutrient absorption capacity of the root genotype used in the experiment. Given that plants already possess an adequate nutrient supply, even if mycorrhizae further improve nutrient uptake, there is no need for plants to activate the phosphate transporter gene. Additionally, when overall alkaline phosphatase activity is low, detecting significant differences in the expression of the phosphate transporter gene at the transcriptional level becomes challenging.

### 5.4 Exogenous ABA Can Alleviate the Inhibitory Effects on Mycorrhizal Function in ABA‐Deficient Mutants and Under Low pH Conditions

Our results have demonstrated that exogenous ABA can enhance mycorrhizal colonization in mutants and alleviate developmental and functional disorders caused by low pH. Compared with wild‐type, the mycorrhizal colonization of ABA‐deficient mutants was significantly reduced (Figure 6A,D,E), along with the significant inhibition of alkaline phosphatase activity (Figure 8A–E). These findings indicate that ABA deficiency substantially suppresses the development and function of mycorrhizae. However, upon supplementation with exogenous ABA in ABA‐deficient mutants, both the mycorrhizal colonization (Figure 6A–C) and alkaline phosphatase activity (Figure 8A,E) were significantly increased, suggesting that exogenous ABA effectively restores mycorrhizal colonization and function (Figure 6).

This conclusion is further supported by previous studies that ABA is a key regulator in the infection process of AM fungi. For instance, the studies of Aroca et al. (2008) and Martín‐Rodriguez et al. (2010) indicated that in ABA‐deficient mutants, the infection ability, the formation process of arbuscules and their functions of AM fungi were all significantly inhibited. Moreover, exogenous ABA application partially restored mycorrhizal colonization, arbuscule development, and function in mutants, whereas sodium tungstate (an ABA synthesis inhibitor) markedly suppressed these processes. Under low pH conditions, the proportion of functional alkaline phosphatase (F‐ALP%, M‐ALP%, m‐ALP%, A‐ALP%, a‐ALP%) decreased significantly; however, after applying ABA, no significant differences were observed across different pH levels. This suggests that exogenous ABA treatment effectively alleviates the inhibitory effects of low pH on mycorrhizal function, thereby restoring mycorrhizal functionality in ABA‐deficient mutants (Figure 8).

### 5.5 ABA May Regulate the Development of Mycorrhiza by Influencing the Synthesis and Transportation of Lipids

Arbuscules are highly branched, tree‐like structures that form during the symbiotic interaction between plants and AM fungi (Luginbuehl and Oldroyd 2017). During the development of arbuscules, they progressively invaginate into the root cortical cells, and the plasma membrane tightly enwraps their structures. The formation of this characteristic arbuscular structure is accompanied by the continuous expansion of the root cell and fungal membrane, thereby creating an extensive specific surface area for substance and signal exchange between the host plant and AM fungi (Bapaume and Reinhardt 2012; Cargill et al. 2025; Perotto and Balestrini 2024). The development of arbuscules involves the formation of numerous membrane structures enriched in lipids, necessitating substantial fatty acid synthesis and transport (Luginbuehl and Oldroyd 2017; Wang et al. 2017; Wipf et al. 2019).

Wang et al. (2012) were the first to identify that the plant gene RAM2 plays an essential role in arbuscule formation. *RAM2* encodes 3‐phosphoglycerol acyltransferase, which participates in lipid biosynthesis within the endoplasmic reticulum. Subsequent research has demonstrated that lipid transfer from plants to AM fungi or pathogenic fungi also requires *RAM2* (Wang et al. 2012). Additionally, *STR2* encodes an ATP‐binding cassette transporter located in the PAM, which is critical for arbuscule formation. Jiang et al. (2017) further elucidated that *STR* and *STR2* are responsible for facilitating lipid transfer from the host plant to AM fungi. These findings underscore the pivotal role of host‐derived lipids in arbuscule formation.

The results of this study show that in the presence of ABA, the expression levels of *RAM2* and *STR2* in the root MT were significantly increased, while pH had no significant effect on the expression of these two genes. Therefore, the increase in the expression levels of *RAM2* and *STR2* can be attributed to the action of ABA. In addition, the addition of ABA significantly promoted the number of arbuscules and the relative arbuscular abundance in mycorrhizal colonization, while pH had no significant effect on the relative arbuscular abundance. Given that *RAM2* and *STR2* are key genes for lipid synthesis and transportation during the process of arbuscule formation, this indicates that ABA may regulate the development of mycorrhiza by regulating the synthesis and transportation of lipids (Figure 9).

### 5.6 Low pH Affects Arbuscule Formation and Function by Regulating ABA in Roots

Our results demonstrated that low pH significantly suppresses the formation of arbuscules, whereas exogenous ABA can enhance their formation. Furthermore, exogenous abscisic acid greatly alleviated the inhibitory effect of low pH on mycorrhizal function. To further investigate whether low pH inhibits arbuscule development and function by reducing ABA content in plant mycorrhizae, we measured ABA levels in mycorrhizal roots under various treatment conditions. The findings reveal that in wild‐type roots, low pH significantly decreases ABA content in mycorrhizae, while the application of exogenous ABA significantly elevates ABA levels in mycorrhizae. This change is positively correlated with the impact of low pH on arbuscule development and function, suggesting that in wild‐type roots, low pH may inhibit arbuscule development and function by suppressing ABA content in mycorrhizae.

Furthermore, it was observed that in commonly cultivated varieties, low pH transiently reduced ABA concentration, with this effect limited to 4 WAI (Figure 3). In contrast, wild‐type MT exhibited a significant and sustained reduction in ABA content following prolonged cultivation under low pH conditions (12 WAI). This discrepancy may be attributed to genotypic differences in sensitivity to environmental stressors. The results demonstrate that low pH significantly suppresses endogenous ABA levels. In this study, exogenous ABA was applied, leading to a marked increase in endogenous ABA concentration, indicating that externally supplied ABA effectively influences internal hormone concentrations. The duration of exposure to exogenous ABA appears to have a persistent regulatory effect on endogenous ABA levels. Notably, a significant interaction between exogenous ABA application and low pH conditions was detected in modulating endogenous ABA concentration. Following long‐term cultivation, treatment groups continued to exhibit distinct ABA profiles, underscoring the lasting impact of these factors.

## 6 Conclusions

This study reveals the key mechanism by which exogenous ABA regulates the efficiency of AM symbiosis under low pH conditions, providing a new theoretical basis and potential strategies for alleviating acid red soil stress and enhancing crop productivity. Firstly, a low pH environment significantly inhibits the colonization of AM fungi and the formation of their key structure—arbuscules. However, the application of an appropriate concentration of exogenous ABA can effectively reverse this inhibitory effect, not only promoting mycorrhizal development but also significantly enhancing the physiological functions of AM fungi, especially in ABA‐deficient plants. Secondly, the mechanism study shows that ABA mediates the development and functional maintenance of arbuscules by regulating the lipid biosynthesis and transport pathways in the host plant. Meanwhile, low pH stress indirectly affects the establishment and efficiency of mycorrhizal symbiosis by disrupting the endogenous ABA homeostasis in the root system. These results clarify the core regulatory role of ABA in plant‐microbe interactions at the metabolic and signaling levels. Furthermore, this study is the first to discover significant genotypic differences in response to low pH stress in the tomato hairy root system, providing important genetic resources and screening criteria for the selection and breeding of crop varieties adapted to acidic soils.

In conclusion, this study not only deepens the understanding of the physiological and molecular mechanisms of ABA signaling in AM symbiosis responses to acid stress but also provides a new perspective for enhancing the ecological function and production potential of acidic red soil agricultural systems through the coordinated regulation of microorganisms and plants. In future work, further analysis of the synergistic stress resistance mechanisms of different crop genotypes and AMF combinations, as well as the application research of ABA analogs or mycorrhizal bioagents in field practice, will contribute to the sustainable development of agriculture in red soil regions and the dual improvement of ecological and production benefits.

## Funding

This work was supported by the National Key Research and Development Program of China, 2023YFF1000300. Natural Science Foundation of China, 32200087, 42077040. Science and Technology Program of Guangzhou, 2024A04J5014. GDAS' Project of Science and Technology Development, 2022GDASZH‐2022010101. The Department of Education of Guangdong Province, 2019GKQNCX067.

## Conflicts of Interest

The authors declare no conflicts of interest.

## Supporting information


**Data S1:** pei370124‐sup‐0001‐Supinfo.pdf.

**Data S2:** pei370124‐sup‐0002‐DataS1.xlsx.

## Data Availability

The original contributions presented in the study are available for free to support transparency and reproducibility in scientific research. For detailed data, please refer to the data sharing file provided in the Supporting Information—S1.
