# Supplementary material for: Low Dosage of ABA Enhances Arbuscule Formation and Recovers the Inhibitory Effect of Low pH on This Process
Source: Plant Environ Interact. 2026 Apr 1;7(2):e70124. doi: 10.1002/pei3.70124 (PMC13042966; doi:10.1002/pei3.70124)
Supplement: Supplementary file 1 — Data S1: pei370124‐sup‐0001‐Supinfo.pdf. [file PEI3-7-e70124-s001.pdf]

Supplementary Material

Supplementary Table

Table1 Standard curve and retention time for ABA

Phytohormone	Standard curve	Time of appearance（min）
ABA	Y=12.119x-0.53;R2=1	29.59

Supplementary Figures

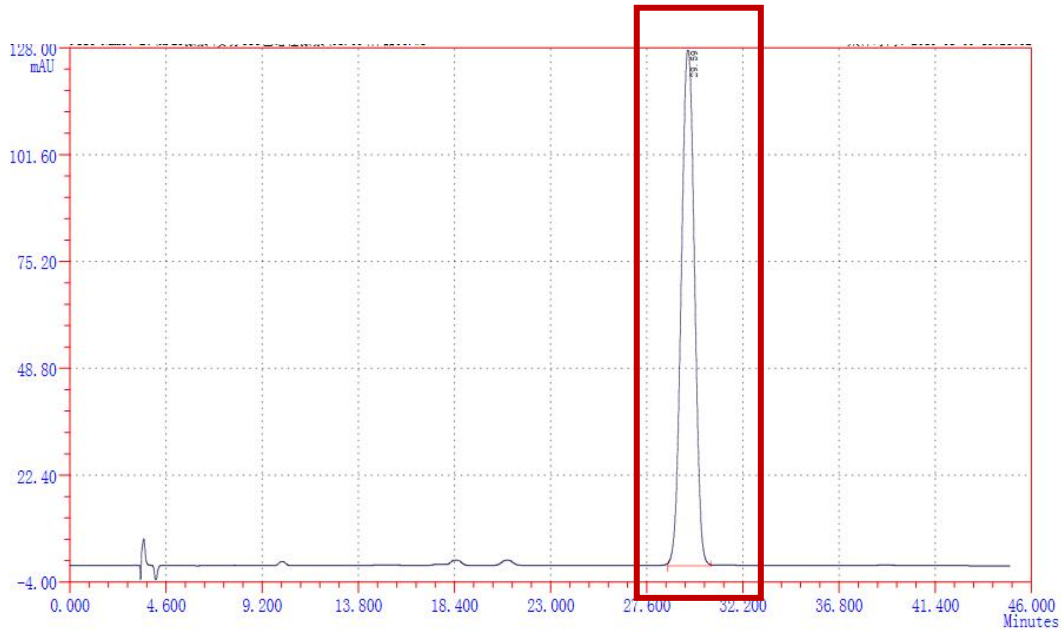

Supplementary Figure1 Chromatogram of the ABA standard. The peak of ABA standard is located within the red rectangular box.

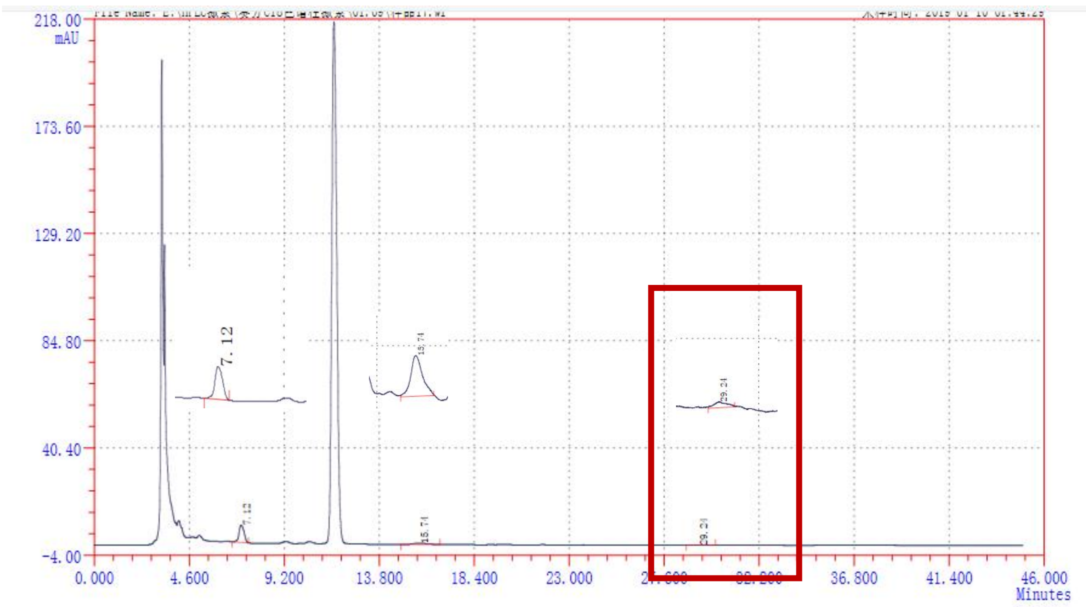

Supplementary Figure2 Experimental sample Chromatogram of the ABA. We also determined the levels of two additional plant hormones (with retention times of 7.12 min and 15.74 min), which were not relevant to the findings of this study and can therefore be disregarded. The peak of ABA in Experimental sample is located within the red rectangular box.

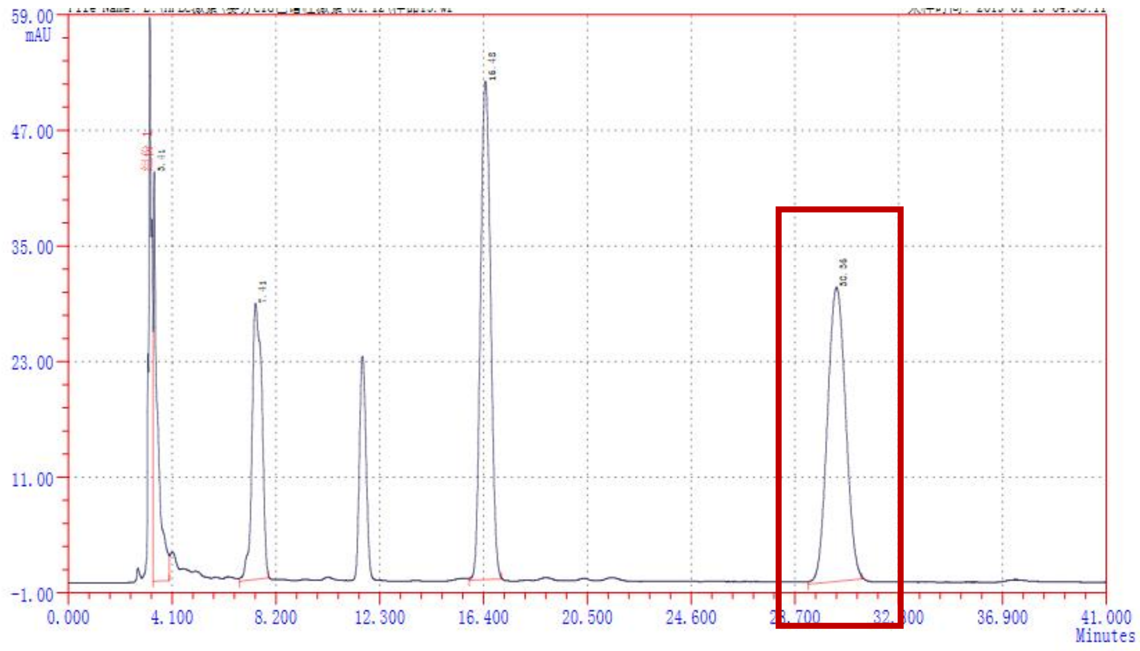

Supplementary Figure3 Chromatogram of the spiked sample. We also determined the levels of two additional plant hormones, which were not relevant to the findings of this study and can therefore be disregarded. The peak of ABA in Experimental sample is located within the red rectangular box.

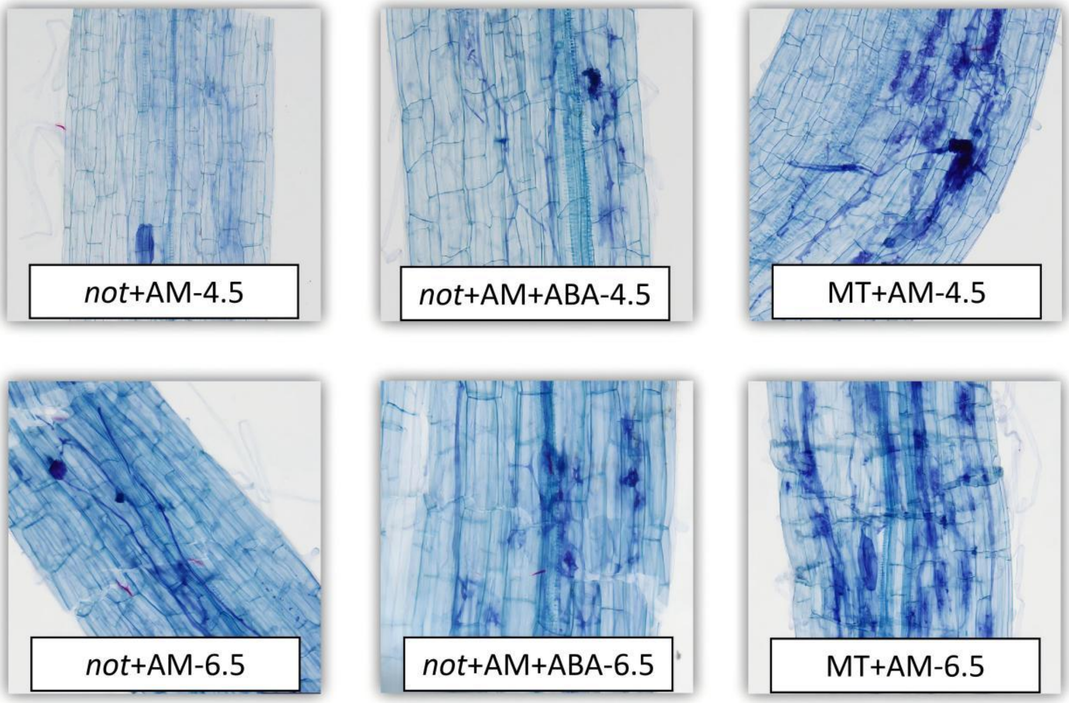

Supplementary Figure4 Mycorrhizal colonization figures of wild-type (MT, Micro-Tom) and ABA-deficient mutant (*not*, *notabilis*) tomato hairy roots under different pH and ABA treatments. -4.5 and -6.5 respectively represent pH 4.5 and pH 6.5 treatments; +ABA and -ABA respectively denote the treatments with and without exogenous ABA addition; +AM indicates the treatment with AM fungi inoculation.

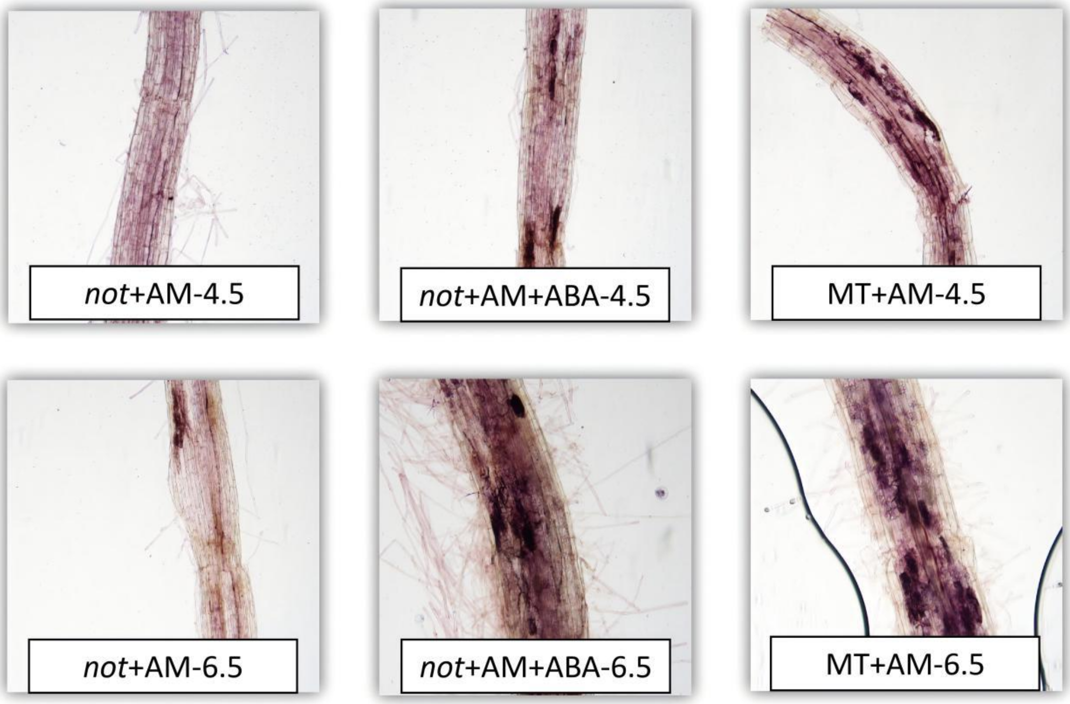

Supplementary Figure5 Alkaline phosphatase activity figures in wild-type (MT, Micro-Tom) and ABA-deficient mutant (*not*, *notabilis*) tomato hairy roots under

different pH and ABA treatments.4.5 and -6.5 respectively represent pH 4.5 and pH 6.5 treatments; +ABA and -ABA respectively denote the treatments with and without exogenous ABA addition; +AM indicates the treatment with AM fungi inoculation.
